# Blood-Based Biomarkers in Hepatitis B Virus-Related Hepatocellular Carcinoma, Including the Viral Genome and Glycosylated Proteins

**DOI:** 10.3390/ijms222011051

**Published:** 2021-10-13

**Authors:** Sanae Hayashi, Katsuya Nagaoka, Yasuhito Tanaka

**Affiliations:** Department of Gastroenterology and Hepatology, Graduate School of Medical Sciences, Kumamoto University, Kumamoto 860-8556, Japan; sanaehayashi66@gmail.com (S.H.); knagaoka@kumamoto-u.ac.jp (K.N.)

**Keywords:** hepatitis B virus (HBV), hepatocellular carcinoma (HCC), HBV genotype, HBV genetic mutations, hepatitis B core-related antigen (HBcrAg), alpha fetoprotein-L3 (AFP-L3), Mac-2 binding protein glycosylation isomer (M2BPGi)

## Abstract

Hepatitis B virus (HBV) infection is a major risk factor for hepatocellular carcinoma (HCC) development and is a global public health issue. High performance biomarkers can aid the early detection of HCC development in HBV-infected individuals. In addition, advances in the understanding of the pathogenesis of HBV infection and in clinical laboratory techniques have enabled the establishment of disease-specific tests, prediction of the progression of liver diseases, including HCC, and auxiliary diagnosis of HCC, using blood-based methods instead of biopsies of liver or HCC tissues. Viral factors such as the HBV genotype, HBV genetic mutations, HBV DNA, and HBV-related antigens, as well as host factors, such as tumor-associated proteins and post-translational modifications, especially glycosylated proteins, can be blood-based, disease-specific biomarkers for HCC development in HBV-infected patients. In this review, we describe the clinical applications of viral biomarkers, including the HBV genome and glycosylated proteins, for patients at a risk of HBV-related HCC, based on their molecular mechanisms. In addition, we introduce promising biomarker candidates for practical use, including colony stimulating factor 1 receptor (CSF1R), extracellular vesicles, and cell-free, circulating tumor DNA. The clinical use of such surrogate markers may lead to a better understanding of the risk of disease progression and early detection of HCC in HBV-infected patients, thereby improving their prognosis.

## 1. Introduction

Two billion people in the world are estimated to have been infected with hepatitis B virus (HBV) and approximately 260 million of them remain persistently infected (hepatitis B surface antigen (HBsAg)-positive HBV carriers). The number of deaths due to liver cirrhosis and liver cancer caused by HBV infection is estimated to be 887,000 per year [1]. Plummer et al. estimated that there are 420,000 cases of HBV-related carcinogenesis worldwide, accounting for 19.2% of all infection-related carcinogenesis [2]. Most hepatocellular carcinomas (HCC) in patients infected with HBV develop after progression of liver fibrosis. On the other hand, carcinomas sometimes occur in cases with histologically mild hepatic fibrosis, suggesting that there are multiple pathways underlying the development of HCC caused by HBV.

The National Institutes of Health (NIH) National Cancer Institute (NCI) defines a biomarker as “a biological molecule found in blood, other body fluids, or tissues that is a sign of a normal or abnormal process, condition, or disease” (https://www.cancer.gov/publications/dictionaries/cancer-terms/def/biomarker) (Accessed on 12 October 2021) [3]. We emphasize that a good clinical biomarker for patients should be (1) simple to test and minimally invasive, (2) highly disease-specific, and (3) highly sensitive. Good candidates for biomarkers for HCC are disease-specific molecules, such as nucleic acids and proteins released from HCC cells into bodily fluids that are relatively easy to collect, such as blood, saliva, and so on.

The Biomarkers for HCC are required to play several roles. Firstly, early detection of HCC development (including suspicion of HCC), secondly, prediction of the progression of HCC, and thirdly, adjunctive role for the definitive diagnosis of HCC. Japanese clinical practice guidelines for HCC propose the use of serum biomarkers, alpha-fetoprotein (AFP), and protein induced by vitamin K absence or antagonist-II (PIVKA-II, also known as des-γ-carboxy prothrombin (DCP)), for routine follow-up of patients with chronic HBV infection [4]. In patients with elevated levels of these serum biomarkers, the suspection of HCC can be diagnosed by detailed imaging studies or sometimes tumor biopsy. Surveillance using minimally invasive, high-performing biomarkers offers significant clinical benefits to patients who need screening for early HCC, if the cost is not a consideration. In addition to the role in the early detection of HCC, biomarkers have another role in identifying patients at high-risk of HCC who need frequent imaging surveillance. Therefore, the development of superior biomarkers and the pursuit of biomarker performance will contribute directly to improving the prognosis of patients with liver cancer. Recently, new biomarkers for liver diseases, based on post-translational modifications by glycosylation, have been attracting attention. In addition to tumor markers for HCC, the HBV genotype, HBV genomic mutations, host genome mutations in HBV-infected hepatocytes and gene expression patterns of HCC cells could be candidate biomarkers for HBV-related HCC [5,6,7]. Novel technologies including these biomarkers have the potential to predict the prognosis of HCC patients. They also potentially enable identification HCC at a very early stage without the need for imaging studies, or diagnose HCC as a surrogate for tumor biopsy. In this review, we summarize novel blood-based biomarkers for HBV-related HCC, especially those focused on mutations in the HBV genome and glycosylation during hepatocarcinogenesis.

## 2. Carcinogenesis of Hepatocytes Infected with HBV

Many cases of chronic hepatitis B progress to cirrhosis and then to hepatocellular carcinoma [8]. The molecular mechanisms of carcinogenesis from chronic inflammation by HBV share a number of common pathways with those from chronic hepatitis C. They reportedly involve accumulation of DNA damage, genomic/epigenetic alterations [9], oxidative stress [10] and cellular senescence [11]. As discussed below, HBV is classified into nine genotypes, and the clinical characteristics are known to vary, depending on the genotype. For example, genotype C is associated with a high rate of progression to cirrhosis, compared to genotype B [12,13]. Taiwanese groups reported that the baseline HBV DNA level is an independent predictor of HCC risk [14] and that the subset with high HBsAg levels is associated with an increase in the incidence of liver carcinogenesis, among the group with low HBV DNA levels [15].

On the other hand, carcinogenesis in young people who are inactive carriers, those with chronic hepatitis and mild fibrosis, and even patients being treated with nucleos(t)ide analogs (NAs), are problems for surveillance because of the difficulty of predicting HCC development. The viral genome persists in the HBV-infected cells in the form of covalently closed circular DNA (cccDNA) outside the host chromosome. The HBV genome encodes protein X (HBx), which promotes the transcription of the viral genome. Studies using transgenic mice suggested that overexpression of HBx may be involved in the development of liver cancer through the promotion of DNA synthesis [16]. The integration of HBV DNA fragments into the host chromosome is often observed in HBV-related liver cancer tissue [17], and the integrated DNA frequently includes the HBx gene [18]. A fusion protein expressed from the HBx gene, integrated into the host DNA, has transactivation function, through which it activates host gene expression, and this is believed to be the cause of hepatocarcinogenesis.

Once HBV infection is established, it cannot be completely eliminated from liver cells. Treatment is aimed at a so-called “functional cure”, which means loss of HBsAg and a decrease in the amount of cccDNA, the stable, extra-chromosomal transcriptional template of HBV. Treatments for chronic hepatitis B infection are injections of interferons (IFNs) or antiviral medications using NAs. NAs significantly inhibit the replication of HBV DNA, making it difficult to check changes in HBV DNA level in the blood during treatment. Serum hepatitis B core-related antigen (HBcrAg) reflects the amount of cccDNA and transcriptional activity in hepatocytes and is, therefore, a useful marker for monitoring patients with chronic hepatitis B during treatment with NAs.

## 3. HBV Genotypes

**Figure 1:**
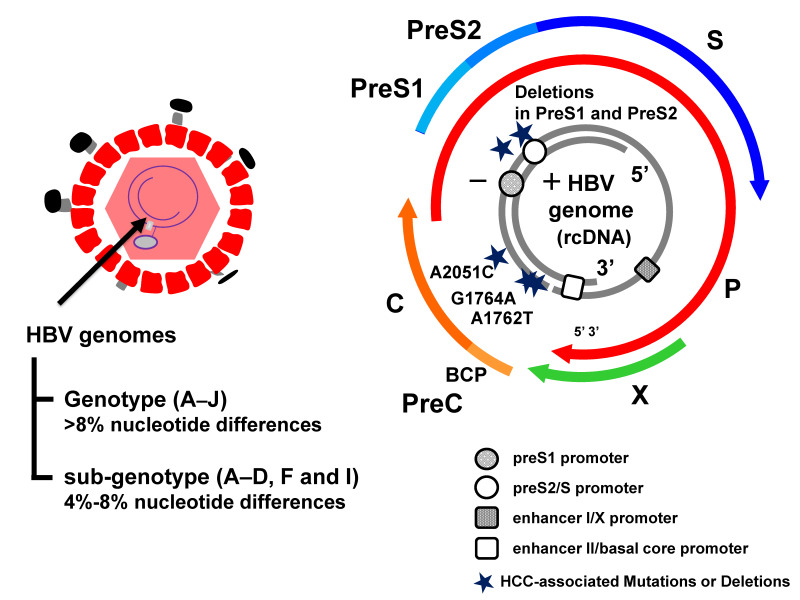
Hepatitis B virus (HBV) mutations associated with hepatocellular carcinoma (HCC). HBV genome and the HBV mutations associated with hepatocarcinogenesis. HBV genotypes (A–J, type I is a subtype of type C) and sub-genotypes (A–D, F, and I) have been classified based on the divergence of the HBV genomes more than 8% and 4%, respectively. The partially double-stranded DNA (dsDNA) encoding four open reading frames (ORFs) and the BCP promoter are shown. ORFs: pre-core/core (preC/C) that encodes the e antigen (HBeAg) and core protein (HBcAg); P for polymerase (reverse transcriptase), PreS1/PreS2/S for surface proteins [three forms of HBsAg, small (S), middle (M), and large (L)] and X for a transcriptional trans-activator protein. The preS1 and preS2/S promoter regions are denoted by the rounds. The enhancer I/X promoter and enhancer II/basal core promoter regions are denoted by the rectangles.

HBV genomes have been categorized into nine distinct genotypes (A–J, type I is a subtype of type C), based on a divergence of more than 8%, and the A–D, F, and I genotypes are further classified into sub-genotypes, based on a divergence of more than 4% [19,20,21] (Figure 1). HBV genotypes/sub-genotypes have distinct geographic distributions and different clinical courses in infected patients [22]. Genotype A is prevalent in Africa, India, North Europe, and North America. Perinatal transmission of sub-genotype A1 in Africa is associated with HCC development in young patients, whereas sub-genotype A2 in Europe and North America is sexually transmitted in adulthood and the infection may persist [23,24]. Genotypes B and C are predominant in the Asia-Pacific region [25] through perinatal or vertical transmission. Sub-genotype B1 is found in Japan and is the most asymptomatic genotype. In contrast, sub-genotypes B2-B5, that are recombinants with the pre-core/core region of genotype C, are similar in their clinical characteristics to HBV genotype C [26]. Previous studies reported that genotype B is associated with earlier HBeAg seroconversion (approximately 10 years) than genotype C, leading to a positive outcome [27,28,29]. Genotype C, found in Southern Asia, including Japan and Hong Kong, has the most sub-genotypes, 16, suggesting their ancient origin [30,31]. Serum HBV DNA levels are higher in patients infected with HBV genotype C1/C2, and especially C4 (a recombinant of genotype C and J), than those with genotype B, resulting in a higher risk of HCC [32]. Sugiyama et al. provided strong evidence that HBV genotype C with high replication efficiency induces fibrosis, with induction of oxidative stress, using chimeric mice with human hepatocytes, which supported the hypothesis that infection with HBV genotype C leads to significantly enhanced disease progression, compared to other genotypes [33,34]. Genotype D is predominant in Africa, Southern Europe, the Middle East, and Oceania, while genotype F is found in Southern and Central America and Alaska [35]. In particular, HBV genotype F1b infection in Native Alaskans is associated with the HCC development at a younger age than other genotypes [36]. Genotype G has been identified in patients co-infected with another genotype, A2 or H, and HIV in France, Germany, and the United States. HBV genotype G, which is incapable of expressing hepatitis B e antigen (HBeAg), expresses competent HBeAg in co-infection with HBV genotype A2 or H [37,38].

These clinical and virological findings indicate that the HBV genotypes exhibit differences in their pathogenicity. However, the significant interaction between HBV and host factors remains to be elucidated, due to the limited experimental models. Therefore, further molecular evidence using multiple in vitro and in vivo approaches is essential to clarify the clinical significance of HBV genotypes.

## 4. Mutations to the HBV Protein Coding Regions and Pathogenesis

**Figure 2:**
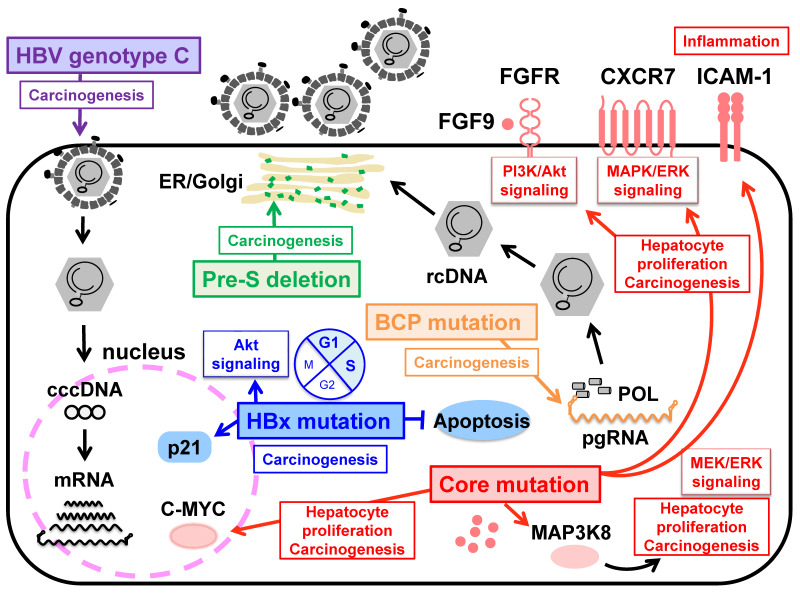
Schematic representation for hepatocellular carcinogenesis associated with HBV pre-S/BCP/Core/X mutations and HBV genotype C. The deletion in the PreS1 and preS2 region may cause overproduction and accumulation of mutated L protein in the endoplasmic reticulum, resulting in inducing HCC development. BCP mutations at nucleotides A1762T and G1764A associated with HBV e antigen (HBeAg) seroconversion and enhancing viral replication is important for carcinogenesis. HBx mutations may regulate apoptosis and transcription indirectly by acting on host cell pathways. The core A2051C mutation in genotype F1b enhancing viral replication mediates in up-regulation/activation of host cell pathways, leading to hepatocellular carcinogenesis. HBV genotype C with high replication efficiency induces oxidative stress and fibrosis, which contributes to hepatocellular carcinoma.

The HBV genome is comprised of four overlapping open reading frames (ORFs) which encode seven proteins (pre-S1, pre-S2, S, pre-C, C, viral polymerase, HBx protein) and there are four regulatory elements (enhancer II/basal core promoter, preS1 promoter, preS2/S promoter, and enhancer I/X promoter) (Figure 1). HBV genetic mutations are detected with high frequency in the basal core promoter (BCP)/pre-core region and the preS/S region [39] (Figure 2). In this section, we describe the emergence of these specific mutations, the pathogenesis, and the clinical consequences of HBV infection. The G1896A mutation in the preC region suppresses HBeAg expression by converting a tryptophan codon (TGG) to a termination codon (TAG) and is the most common mutation in HBeAg-negative patients [40]. The preC region overlaps the epsilon signal, which is essential for pregenomic RNA (pgRNA) packaging, and the G to A substitution improves the U1858-G1896 base pairing in genotypes B, C1, D, E, F2, F3, and H, but disrupts the existing C1858 G1896 base pairing in genotype A and F1 [41,42]. Several meta-analyses revealed that the G1896A mutation, enhancing HBV replication, correlates with an increased risk of acute and chronic liver failure, but not HCC development [43,44]. Our in vitro experiments confirmed the G1896A mutation enhances HBV replication [34,45]. Moreover, a recent prospective study showed that the precore (PC) mutation in HBeAg-positive patients results in rapid HBeAg clearance [46]. From these findings, long-term observations are necessary to determine the impact of the timing of acquisition of the PC mutation on the progression of the disease.

In contrast, the A1762T/G1764A double mutations in the BCP region are associated with cirrhosis and HCC development, irrespective of the HBV genotype [13,47]. The BCP mutations reduce the expression of pre-core RNA to decrease HBeAg expression but also enhance the expression of pgRNA to increase viral replication [48] (Figure 2). These effects of the BCP mutations are further enhanced by the presence of T1753C, C1766T, and T1768A mutations in the BCP region [49,50], suggesting that acquiring these mutations with high replication capacity is important for carcinogenesis. Recently, Hayashi et al. demonstrated that the specific A2051C mutation in the core region enhances HBV replication by stabilizing core protein dimerization in HBV genotype F1b. In human liver chimeric mice infected with HBV with a combination of A2051C and the BCP and PC mutations, upregulation/activation of pathways that may lead to fibrosis and carcinogenesis reveals the potential of carcinogenesis by the A2051C mutation [51] (Figure 2). The BCP region overlaps the X ORF. Since a significant accumulation of HBx proteins is observed in the tumor tissues of HCC patients, the mechanism by which HBx proteins affect the pathogenesis of hepatocellular carcinoma has long been studied [16,52]. Several studies reported that the combination of aaK130M + V131I (corresponding to A1762T/G1764A mutations), with or without I127T, F132Y, and V5M in the X region, is associated with disease progression to HCC development, and the aaK130M + V131I promotes HCC by activating Akt signaling in a transgenic mouse model [53,54,55]. Recently, a unique combination of A10R and S144R was identified in the cancer tissues of HCC patients, and an in vitro study revealed that these double mutations increase *p21* expression, prolong the G1/S transition, and inhibit cell apoptosis [56]. Although HBx proteins play a central role in hepatocarcinogenesis by activating the promoters and enhancers of HBV, as well as host genes, the exact mechanism by which HBx mutations induce HCC remains to be elucidated and requires continued research. Many researchers have investigated the association between deletions in the preS region and liver diseases such as acute hepatitis B, fulminant hepatitis due to HBV infection, chronic hepatitis B infection, liver cirrhosis (LC), and HCC [57,58,59]. In particular, a pre-S deletion of the C-terminal half (aa 58–119) of the preS1 region was frequently observed in CH and LC patients [60,61], and a deletion at the *N*-terminus (aa 1–23) of the preS2 region was common in HCC patients [57,62]. These mutations result in overproduction of the L protein compared to the M and S proteins [63,64]. Deletion of preS1 and preS2, resulting in an imbalance in the production of the envelope proteins, may cause excessive accumulation of the L protein in the endoplasmic reticulum, leading to DNA damage and progression of liver disease [65] (Figure 2). Of note, these regions correspond to HLA-restricted B-cell and T-cell epitopes [66], suggesting that HBV mutations may contribute to immune escape.

Among the multiple HBV genotypes, the key genes and regulatory regions are highly conserved [50,67], but HBV reverse transcription lacks a proofreading activity [68], resulting in the acquisition of mutations and dynamic changes in HBV progression. Therefore, understanding the mechanism by which HBV mutations lead to liver disease may be useful for the identification of targets for new therapeutic agents.

## 5. HBcrAg

**Figure 3:**
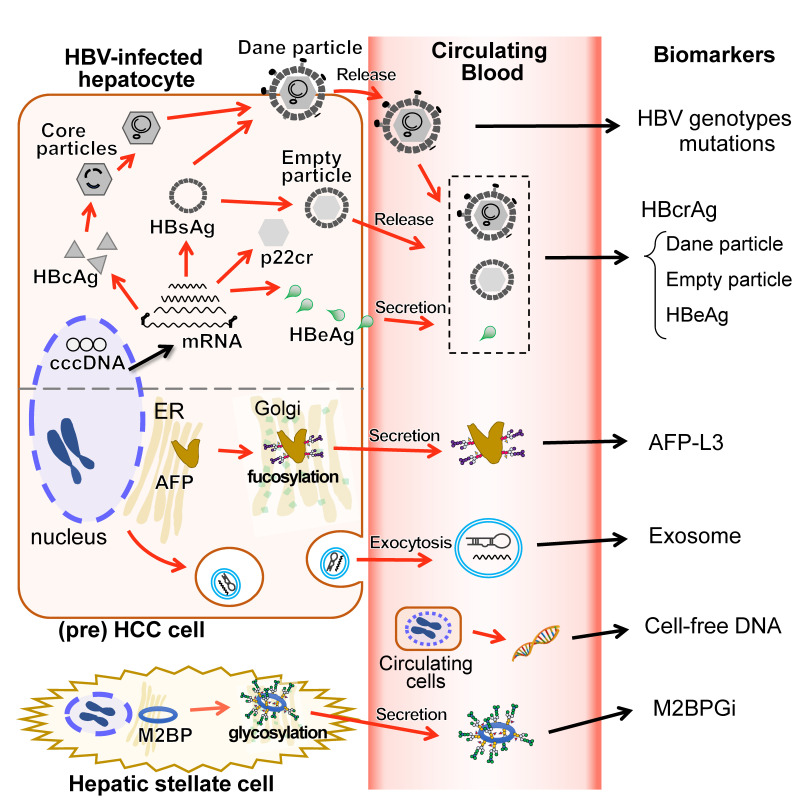
Blood-based biomarkers and their intracellular sources. The schematic diagram shows the link between the blood-based biomarker and the intracellular molecules targeted by each biomarker in HBV-related HCC patients. HBV genotypes/genome mutations are associated with disease progression. HBcrAg consisting of HBcAg, empty particles and HBeAg would correlate to cccDNA, that higher persistent HBcrAg levels are associated with the risk of HCC development with HBV infection. Dane particles refer to infectious complete virions, which have HBcAg inside them. HbeAg crosses the hepatocyte membrane. Both empty particles and HBeAg are released or secreted into the bloodstream in the processes separate from the Dane particles. AFP-L3 is a fucosylated AFP secreted into the bloodstream and can bind to LCA lectin. Fucosylated AFP levels correlates with the poor prognosis of HCC. Exosomes are a subtype of extracellular vesicles that encapsulate microRNA or messenger RNA and are released from cells into the bloodstream by exocytosis. Cell-free DNA in blood derived from the broken circulating HCC cells could be useful for an early detection of HCC. M2BPGi is a M2BP glycosylation isomer and is thought to be secreted from hepatic stellate cells. M2BPGi levels are reportedly associated with the risk of HCC development with HBV infection. HBV, hepatitis B virus; HCC, hepatocellular carcinoma; HBcrAg, hepatitis b core-related antigen; HBsAg, hepatitis B surface antigen; HBcAg, hepatitis B core antigen; p22cr, 22-kDa pre-core protein; HBeAg, hepatitis B e antigen; cccDNA, covalently closed circular DNA; AFP, alpha-fetoprotein; LCA, Lens culinaris (Lentil) Agglutinin; mRNA, messenger RNA. M2BP, Mac-2 binding protein.

Hepatitis B core-related antigen (HBcrAg) consists of three proteins coded by the pre-core/core ORF, hepatitis B core antigen (HBcAg), a 22-kDa pre-core protein (p22cr), and HBeAg [69]. HBcAg proteins are included in infectious complete virions called Dane particles. Apart from these, empty particles (particles without viral DNA) containing HBcAg, p22cr antigen, and HBsAg are formed and released from the hepatocytes. Additionally, HBeAg crosses the hepatocyte membrane and are secreted into the bloodstream. Both empty particles and HBeAg are released from the hepatocytes in the separate processes from the Dane particles (Figure 3). In 2002, Kimura et al. developed a highly sensitive enzyme immunoassay (EIA) specific for HBcrAg [70]. HBcrAg has been commercialized as an HBV marker in Asia and, more recently, in Europe [71]. The HBcrAg assay can detect antigen even in anti-HBc or anti-HBe positive patients, regardless of pre-core mutations [69], and HBcrAg levels correlate with HBV DNA levels in serum and liver, as well as cccDNA in the liver [71,72]. HBV DNA assays are used in clinical practice, but their costs and long measuring times limit their use in various regions [73]. In addition, intrahepatic cccDNA assays require invasive liver biopsies, which make continuous monitoring difficult. Therefore, the significance of HBcrAg measurement as a reliable, easy-to-use, and low-cost assay for the management of CHB patients has been widely assessed [74].

Among naïve CHB patients, serum HBcrAg levels are significantly higher in HBeAg-positive than in HBeAg-negative and are correlated to not only serum HBV DNA levels, but also intrahepatic HBV DNA and pgRNA, besides both intrahepatic cccDNA levels and its transcriptional activity [75]. Moreover, serum HBcrAg levels are reportedly more closely related to intrahepatic cccDNA levels than serum HBsAg and HBV RNA, regardless of HBeAg status [76,77,78], and HBcrAg levels in CHB patients before NA treatment were cited as a predictor of serum HBcrAg levels <3 log10 U/mL(LOD) after eight years of treatment [79]. Long-term monitoring of serum HBcrAg levels is appropriate for CHB patients with undetectable HBV DNA during NA treatment [72], and HBcrAg could be valuable in predicting HBsAg loss and the risk of HBV reactivation after treatment [80]. Many patients receiving NA therapy are recommended to continue treatment, while researchers investigate which cases can safely discontinue the NA therapy. The decision to stop the treatment has been conventionally determined based on viral serological markers, HBV DNA, alanine transaminase (ALT) levels, and serum HBsAg levels. Wong DKH et al. reported that serum HBcrAg levels was significantly correlated with intrahepatic cccDNA levels even during NA therapy [81]. Moreover, in a report by Jung K.S. et al., above 3.7 log IU/mL of serum HBcrAg level after cessation of NA therapy predict virological relapse within a year. [82]. Another report showed that high HBcrAg levels (median; 4.9 log U/mL) after NA cessation can predict the relapse in the patients formerly treated with lamivudine, one of NAs, even if HBV DNA levels are below detection sensitivity for at least six months [83]. Therefore, serum HBcrAg is regarded as one of the useful biomarkers to consider the NA cessation [84]. Similarly, serum HBcrAg in CHB patients before and during PEG-IFN treatment could be an appropriate indicator for achieving HBeAg seroconversion and/or HBV DNA suppression [85,86].

Interestingly, serum HBcrAg levels in CHB patients were also associated with HCC development, regardless of NA treatment [87,88,89,90], and both HBcrAg > 2.9 logs IU/mL and BCP mutations are an independent predictor of HCC in naïve CHB patients [87,88]. In a recent longitudinal analysis, the combination of low HBsAg/high HBcrAg levels was associated with significantly more frequent HCC [91]. Furthermore, high intrahepatic cccDNA and serum HBcrAg levels in HCC patients were significantly associated with worse recurrence-free survival [92]. These reports suggest that HBcrAg is appropriate for screening CHB patients at high risk for HBV reactivation and HCC development.

In 2001, Su et al. reported that serum HBV-RNA could be identified to detect HBV subclinical infection. In recent years, there has been an increasing number of reports on serum HBV RNA levels in CHB patients, and several papers have suggested that serum HBV-RNA levels could be a marker reflected cccDNA transcriptional activity as well as serum HBcrAg levels [93]. In a report in CHB patients who did not receive NA therapy, HBV RNA had a moderately strong correlation with HBV DNA as well as and HBcrAg with and without HBeAg-positive [94]. In HBeAg-negative patients, even when HBV DNA was suppressed under NA therapy, both serum HBV RNA and HBcrAg were shown to be sensitive markers of cccDNA transcriptional activity [95]. On the other hand, although an association between HBV RNA levels and HCC development are associated at the tissue level [96], clear evidence of an association between serum HBV RNA levels and HCC development has not yet been reported [97].

More recently, Inoue et al. demonstrated that a fully automated, high-sensitivity CLEIA for HBcrAg detection (iTACT-HBcrAg; Fujirebio, Inc., Tokyo, Japan), is about 10-fold more sensitive than the conventional HBcrAg assay, and is even more useful for monitoring transcriptional activity of cccDNA and detecting HBV reactivation before the onset of hepatitis [98,99]. Since iTACT-HBcrAg does not require any special technology and provides results within 30 min; it is expected to be used in clinical practice and research in the future.

Collectively, these reports show that serum HBcrAg can predict clinical outcomes, such as HBeAg seroconversion, response to therapy, HBV reactivation, and risk of HCC development [100].

## 6. AFP-L3

Alpha fetoprotein (AFP) is a serum protein that is produced in large quantities by the yolk sac and liver of the developing fetus. Its production declines after birth and is almost absent in adults. Serum AFP levels have been reported to be elevated in the serum of patients with HCC and have been used as a liver tumor marker [101]. On the other hand, AFP increases with hepatocyte regeneration, such as in cases of liver inflammation [102], reducing the specificity of AFP as a tumor marker. AFP is thought to be produced by mature hepatocytes with high proliferative potential, because an immunohistological study revealed that the majority of AFP-expressing cells are positive for proliferating cell nuclear antigen (PCNA) [103].

In order to solve the problem of specificity of AFP, researchers have focused on the disease specificity of the various glycosylated forms of AFP. Glycans are the third biopolymer, after nucleic acids and proteins, and have a chain-like structures consisting of monosaccharides linked by glycosidic bonds. Glycan structures are known to have organ specificity, and cell surface glycan structures are altered by the tissue condition, disease, and cell transformation [102,104]. Analysis of the glycosylation of AFP allows for qualitative assessment of the proteins expressed by tumors, not just their quantity. The presence of characteristic alterations in glycosylation of AFP in the serum in patients with HCC was reported by Breborowicz et al. in 1981 [105]. In 1990, according to the affinity of fucosylated AFP for the Lens culinaris (Lentil) Agglutinin (LCA) lectin, Taketa et al. [106] classified them into LCA-non-reactive (L1), weakly reactive (L2), and strongly reactive (L3) fractions, and showed that the L1 fraction of AFP was increased mostly in chronic hepatitis and cirrhosis, while the L3 fraction was increased in hepatocellular carcinoma.

Some reports, including that by Yamashita et al. [107], suggest that the serum AFP-L3 value alone can predict the prognosis of hepatocellular carcinoma. Which of the three markers, AFP, AFP-L3, and PIVKA-II, is positive varies among cases because of the diversity of HCC, and this trend is particularly clear in early-stage HCCs [108]. Therefore, researchers are attempting to improve diagnostic and prognostic performance by using a complementary combination of the three or other clinical parameters. The GALAD score consists of five parameters: G: gender, A: age, L: AFP-L3, A: AFP, and D: des-gamma-carboxy prothrombin (DCP). The GALAD score is highly objective and has similar usefulness for the detection of hepatocellular carcinoma in different countries [109,110]. The BALAD score and BALAD2 score are indexes created by adding bilirubin and albumin levels to the above three tumor markers. The BALAD score is reportedly useful in predicting the prognosis of HBV-related liver cancer [111] and international joint research, including the United Kingdom and Germany, reported that the BALAD2 score can also predict the prognosis of liver cancer in chronic liver diseases of various etiologies [109].

## 7. M2BPGi

Mac-2 binding protein glycosylation isomer (M2BPGi, also known as WFA^+^-M2BP) is a Mac-2 binding protein (M2BP) with a sugar chain on its surface that is capable of binding Wisteria floribunda (WFA) lectin. M2BPGi was identified by a comprehensive analysis of glycosylation in sera from patients with chronic hepatitis C and reportedly showed a significant correlation between its levels in serum and progression of fibrosis in the patients [112]. As the name implies, M2BP binds to Mac-2 (or galectin 3), a galactose-binding protein expressed by macrophages and Kupffer cells. High M2BP levels in the sera of patients with breast cancer, lung cancer, pancreatic cancer [113], and HCC [114] are reported to correlate with poor prognosis. M2BP forms a ring-shaped multimer in the blood and its large surface area makes it susceptible to glycosylation [115]. Shirabe et al. stated that a source of M2BP is the hepatic stellate cell, based on their immunohistochemical (IHC) analysis [116,117] and fluorescence in situ hybridization [118]. Another analysis shows M2BP to be located on Kupffer cells [116]. Furthermore, stimulation by M2BPGi induces Mac-2 expression by Kupffer cells [116]. Therefore, it was suggested that M2BP secreted from hepatic stellate cells may be taken up by Kupffer cells [117]. Thus, M2BPGi is not only useful as a serum biomarker for prediction of liver fibrosis but also may play an important role in the progression of fibrosis through inflammation in the extracellular matrix of liver tissue [119].

Besides assessing fibrosis in chronic liver disease, serum M2BPGi levels are significantly associated with the risk of HCC development with HBV and HCV infection [120,121,122]. Jun et al. reported that M2BPGi is more effective than AFP in predicting the development of HCC in CHB patients [123]. In CHB patients with NA treatment and in cases with undetectable HBV DNA, patients with high levels of M2BPGi before treatment are at increased risk of developing HCC [124,125].

## 8. Other Biomarkers

Assessment of the blood biomarkers discussed above has already reached a consensus in the scientific community. Here, we shall briefly describe some of the candidates that may be expected to be developed as promising blood biomarkers for HBV-related liver cancer in the future.

### 8.1. WFA^+^-CSF1R

As described above, comprehensive analysis of glycosylation in sera from patients with chronic hepatitis led to the evaluation of M2BPGi as a serum biomarker for liver fibrosis. Another glycan marker for HCC, WFA^+^-colony stimulating factor 1 receptor (CSF1R), was identified from the analysis of sera from patients with chronic hepatitis C and cirrhosis [126]. CSR1R is a membrane protein with functions related to macrophage differentiation and cell proliferation. CSF1R expression in tumor tissues reportedly correlates with poor prognosis in breast, prostate, and ovarian cancers. An analysis of sera from patients with chronic hepatitis C, cirrhosis and HCC showed that WFA^+^-CSF1R is potentially useful for predicting liver carcinogenesis and prognosis of cirrhosis [127].

### 8.2. Extracellular Vesicles

Extracellular vesicles (EVs) are particles delimited by a lipid bilayer and covered with sugar chains, and are secreted by cells into the bodily fluids [128]. EVs are classified into several types, such as exosomes, microvesicles, and apoptotic vesicles, by their origin, size, and other characteristics, however, it is sometimes difficult to make a strict distinction. Exosomes are vesicles formed during the endocytotic process and are known to encapsulate cell-derived nucleic acid fragments, such as microRNAs (miRNAs). Exosomes also are found in large amounts in the blood and are expected to be valuable in liquid biopsies. Matsuura et al. analyzed the EVs extracted from the plasma of patients with chronic hepatitis C and found that the levels of miRNAs called let-7 correlated with the progression of liver fibrosis stage [129]. Another report described decreased expression of four miRNAs, including miRNA-192 and miRNA-122, among the miRNAs obtained from EVs in plasma from both HBV-and HCV-infected patients, compared to healthy subjects [130].

Analyses of sera from patients with chronic liver disease type B showed that the expression of several miRNAs (miR-21 [131,132], miR-18a, miR-221, miR-222 and miR-224 [133]) and messenger RNA hnRNPH1 expression [134] in EVs were significantly higher in chronic hepatitis B and cirrhosis patients with HCC than chronic hepatitis B and cirrhosis patients without HCC.

### 8.3. Cell-Free DNA/Circulating Tumor DNA

Cell-free DNA (cfDNA) in plasma is mostly thought to be DNA derived from lysed hematopoietic cells [131], while, in cancer patients, it is known that the DNA is partially derived from cancer cells which are destroyed by the immune system or apoptosis. Extracellular DNA in the blood is called “cfDNA”, while DNA derived from isolated tumor cells circulating in the blood is called “circulating tumor DNA (ctDNA)”.

Qu et al. [135] evaluated the ability of a screening test to identify early HCC using cfDNA with somatic mutations in the blood of HBsAg positive, asymptomatic individuals. cfDNA was extracted from the blood and examined for the presence of mutations in at least one of the following genes: *TP53*, *TERT*, *CTNNB1*, *AXIN1*, and *CTNNB1*. In addition, HBV integrations were also selected as genomic aberrations for detection in cfDNA. Among patients who tested negative when screened for HCC by serum AFP level or abdominal ultrasound, the sensitivity (the percentage of patients who tested positive on the cfDNA test and had HCC detected on abdominal contrast-enhanced computed tomography) was 100% (4/4), while the specificity was 94% (307/324). Another report showed that total plasma cfDNA levels could be a biomarker to predict early recurrence of HBV-related HCC in patients after resection of HCC [136].

## 9. Conclusions

In this review, we have described the current status of blood-based biomarkers in HBV-related HCC, based on the molecular mechanisms of the HBV genomic features, mutations, and glycosylated protein from the cells in relation to the pathogenesis of the tumor (Figure 3). The identification of HBV genotype provides some indication of the clinical features and risk of liver carcinogenesis. The mutations in the coding region of the HBV protein provide information on the molecular biological pathogenesis leading to liver disease. HBcrAg levels also reflect the risk of HCC and can be used to monitor for reactivation. AFP-L3 levels are helpful for screening and determining the prognosis of liver carcinogenesis and M2BPGi testing enables the prediction of liver fibrosis and HCC development. All of these biomarkers have already been put to use in clinical practice and all provide information that can be obtained from the patient’s blood.

Novel technologies, including the comprehensive analysis of glycosylation, analysis of extracellular vesicles, and analysis of cfDNA/ctDNA (Figure 3), are expected to be put to practical use in the near future as candidate blood biomarkers with higher sensitivity and specificity. Since the discovery of HBV as ‘Australian antigen’ by Blumberg et al. in 1964, vigorous research by many investigators has greatly advanced the technology for diagnosis and treatment of HBV-related HCC. On the other hand, the number of HBV-infected patients worldwide has been increasing, and we still have much work to do. In order to reduce HBV-related cirrhosis and HCC, further progress in the development of blood-based biomarkers is desired.

## Data Availability

Not applicable.
